# Infantile Convulsions

**Published:** 1889-07

**Authors:** E. M. D. Bridgford

**Affiliations:** St. Louis, Mo.


For Daniel's Texas Medical Journal.

### INFANTILE CONVULSIONS.

BY E. M. D. BRIDGFORD, M. D., ST. LOUIS, MO.

OF ALL THE ILLS and complaints incident to early life there is not, perhaps, any one so full of interest to the general practitioner as convulsions. We are often called to see the only child of young parents, in a spasm, and seemingly near death's door. Convulsions, under any circumstances and at any age, are well calculated to inspire alarm, and we cannot, therefore, be surprised at the anxiety exhibited by the mother in behalf of her infant. The physician who successfully treats the sick baby is most certain to get the family practice, and will always be duly appreciated by the mother.

In children, convulsions are generally symptomatic, and are traceable to a great variety of causes. To enumerate them all in this article is scarcely necessary, especially as it would be a very difficult task. Prof. A. S. Barnes, M. D., of the St. Louis College of Physicians and Surgeons, claims that at least 75 per cent. of all the diseases of children are due to improper feeding. One of the most severe convulsions I ever saw, was the result of feeding a child about eighteen months old, tough beef and fried potatoes. In less than an hour the child was taken with a spasm, which lasted until an emetic was given, and the contents of the stomach ejected.

The powers of assimilation in the child, are not sufficient to appropriate a laboring man's meal to its own use. If an infant is fed food that is hard to digest, a quantity will pass into the bowels undigested, where it will undergo fermentation, and may produce a dysentery, entero-colitis, or some other intestinal disorder. Teething, constipation, and injury to the child during labor, are frequent causes of convulsions.

As convulsions is not a disease, but a symptom of some abnormal condition of one or more of the bodily organs, we should have a thorough knowledge of the physiology of the human organism, and also a good understanding of the pathological changes the organs are likely to make. It is always our duty to find, if possible, the cause of convulsions, and remove it, and not get in the habit, (like some few practitioners,) of giving a hot bath and a little bromide for every case we see. I will now try to report three cases of convulsions, each of which was produced by a different cause:

CASE I.—Fanny S., age fifteen months. Always healthy, except occasionally constipated. At the time she was a year old, she commenced to vomit immediately after nursing. The attending physician recommended a change of food, and she was given diluted cow's milk, instead of the mother's milk, and the child improved for a while. A few weeks later the vomiting returned, with increased violence, and it seemed as if nothing could be given to relieve it. I learned from the mother that the vomit was of a greenish color, and always followed by a convulsion. By a further examination, I found the liver was greatly enlarged, and very painful. The acute hepititis, I think, was the cause of the convulsions. To relieve the vomiting, which was very acid, I gave the following:

| | | |
|---|---|---|
| ℞ | Acid Carbolici | gtt. iv. |
| | Bismuth Sub. Nit. | ʒiiss. |
| | Glycerine | ℥ss. |
| | Aq. Mentha piper q. s. ad | ℥iv. |

M. et Sig.: Teaspoonful before feeding.

For the hepatic engorgement, I prescribed:

| | | |
|---|---|---|
| ℞ | Sodii Bicarb. | gr. x. |
| | Hydrarg Chlor. Mitis. | gr. i. |

M. ft. charts No. 10 et Sig.: One powder every two hours.

Commencing one hour after giving the first powder, I directed a teaspoonful of the following to be given every two hours:

| | | |
|---|---|---|
| ℞ | Kali Iodidi | gr. xvi. |
| | Aq. Destil | ℥iv. |

M. Sig.

I gave the last two prescriptions for three days, and then for the same length of time I did not give any medicine, unless the vomiting returned, and I gave the first mixture. After continuing the above plan of treatment for some time, the child's condition was greatly improved. It died soon afterwards, of bronchitis.

CASE 2.—Anna T., age two years. Had always been healthy until a few days before she was taken with a convulsion. I examined the patient carefully, and found all the organs normal, excepting a slight bronchitis. I could not persuade myself to think that the mild inflammation of the bronchi would cause a convulsion. The child's grandmother said it had been picking its nose for several days, and she thought probably it had worms. I accepted the old lady's diagnosis, which proved to be correct, and prescribed:

| | | |
|---|---|---|
| ℞ | Sodii Bicarb. | gr. xii. |
| | Calomel | gr. i. |

M. ft. chart No. 8 et Sig.: One every hour, the last powder to be followed with a teaspoonful of castor oil, with one drop of turpentine.

The directions were carried out, and as a result the child had eight or ten passages, the last of which contained a pint of small worms. A complete recovery soon followed.

CASE 3.—Oscar E., age eight months. Had been having convulsions for nearly two weeks. The patient was a fat, healthy boy, and there was not the slightest trace of disease, so far as the child was concerned. I requested the mother to send me a small quantity of her milk, which I examined, and found it was of a strong acid reaction. I suggested that the child be denied the breast milk, and be fed with the milk of a healthy cow, and also "Mellin's Food." The result was very favorable, and the child is now doing well, and enjoying the best of health.

